# Editorial: Inflammasomes: Cornerstone of Immunity

**DOI:** 10.3389/fcell.2022.888378

**Published:** 2022-04-14

**Authors:** Cédric Rébé, François Ghiringhelli, Deepika Sharma

**Affiliations:** ^1^ Centre Georges François Leclerc, Dijon, France; ^2^ INSERM U1231 Lipides, Nutrition, Cancer (LNC), Paris, France; ^3^ Committee on Immunology, Chicago, IL, United States

**Keywords:** inflammasomes, immunity, IL-1 (interleukin-1), GSDMD, disease

Inflammasomes are multimeric protein complexes activated in response to a wide range of pathogen- and damage-associated molecular patterns that induce auto-processing of the proenzyme caspase-1. Activation of caspase-1 results in pyroptotic cell death, and the processing and release of the inflammatory cytokines, IL-1β and IL-18, primarily through gasdermin D (GSDMD) pores. A diverse range of sensors, triggers, and regulators makes inflammasome activation a tightly controlled process that is central to health and disease. Dysregulation of these processes is associated with various inflammatory disorders, including sepsis, cancer, cardiovascular diseases, and even neurological disorders.

This Research Topic includes studies identifying and discussing the recent advances in the regulation and function of inflammasome and associated molecules in modulating immunity.

Two papers reviewed the impact of inflammasomes within immune and cancerous cells during tumor development, and how the role of inflammasome is a function of the cell type undergoing inflammasome activation. Andina et al. reviewed the role of inflammasomes in myeloid malignancies including myelodysplastic syndromes, myeloproliferative neoplasms, and acute myeloid leukemia which are heterogeneous disorders originating from genomic mutations in hematopoietic stem and progenitor cells. While the pro-inflammatory environment resulting from Inflammasome activation was shown to be involved in disease initiation and clonal progression, contrary data also showed that inflammasomes can counteract leukemia by mediating cell death in malignant clones. These effects of inflammasome activation were additionally dependent on the mutational context. In myeloid malignancies, controversial roles of inflammasomes were also described in cancer incidence, progression, and response to treatment Saleh et al. described how inflammasomes and more particularly IL-1β or IL-18 promote cancer cell survival, proliferation, and invasion. They can also participate in tissue repair, fibrosis, and immunosuppression. Contrarily, inflammasomes were also shown to maintain tissue homeostasis and to induce anti-tumor immunity. Inflammasome activation can also induce anti-tumor effects in cancer cells by inhibiting their proliferation and promoting their differentiation or pyroptosis, a kind of immunogenic cell death. These studies and the outcomes of trials targeting inflammasomes in cancer highlight that a deeper contextual understanding of the impact of inflammasome activation in the type and the stage of cancers is required to harness the potential of this promising therapeutic target.

Inflammasomes also modulate the incidence or severity of other diseases such as atherosclerosis, bacterial infections, and neurological disorders, such as multiple sclerosis. The role of inflammasomes (NLRP3 or AIM2) and IL-1β in atherosclerosis are well known. Lipid engulfment by macrophages leads to their transformation into foam cells which accumulate in the arterial wall to form atherosclerotic plaques. Removal of excess cholesterol from these cells via Reverse Cholesterol Transport (RCT) may reverse disease progression. While inflammasomes were described to participate in the continued influx of immune cells into the plaques and in the inhibition of RCT, the direct role of GSDMD in atherosclerosis is not fully clear. In their paper, Opoku et al. show that GSDMD mediates inflammation-induced inhibition of RCT and promotes atherosclerosis. Using *Gsdmd* ^*−/−*^ mice, the authors demonstrate that RCT inhibition in response to NLRP3 activation is severely impaired in the absence of GsdmD. This effect is mediated by a secretory factor (such as IL-1β) released through GSDMD pores, as conditioned medium from *Gsdmd* ^*−/−*^ cells is ineffective at inhibiting both RCT and foam cell formation, while WT conditioned medium can inhibit RCT and promote foam cell formation both on WT and *Gsdmd* ^*−/−*^ macrophages. *In vivo*, GSDMD is cleaved in aortic root plaques and participates in RCT suppression. Another impact of GSDMD is to inhibit apoptotic cell death, as *Gsdmd* ^*−/−*^ macrophages seem to be more susceptible to this non-inflammatory cell death. These studies shed light on the various processes modulated by inflammasome activation during the development of atherosclerosis.

Inflammasomes drive pyroptotic cell death and inflammatory cytokine release in response to microbial infections. The amplitude and function of this response depend on the identity of the pathogen. This dichotomous impact of inflammasome activation is illustrated in the study by Guo et al. In this study, the authors used two strains of *Francisella novicida*, the virulent WT (U112) and the 4′-phosphatase-deficient avirulent strain (XWK4) that has an altered lipid A structure. The mutant strain induces higher caspase-1 activation and IL-1β production, through activation of both AIM2 and NLRP3 inflammasomes. However, while inflammasome activation promotes clearance of the WT-U112 *Francisella novicida* strain, it significantly worsens clearance and pathology induced by the mutant XWK4. Further, while the inflammasome activation induced by U112 is dependent on type I Interferons (IFN), XWK4 induced inflammasome activation relies on type II IFN signaling. This work highlights how the engagement of distinct pattern recognition receptors (PRRs), and cell death pathways by pathogens can alter host inflammatory response and outcomes in infectious diseases. Understanding these processes is critical to targeting these complexes to fight bacterial infections.

In addition to understanding the function and context of inflammasome activation, novel and specific inhibitors of the inflammasome pathway are required to modulate these responses in distinct pathological contexts. To address this, Li et al. identified manoalide as a new small molecule inhibitor specific to the NLRP3 inflammasome. It does not impact the proximal processes of potassium and chloride efflux or mitochondrial dysfunction involved in NLRP3 activation. Instead, it covalently binds to Lys 377 of the NLRP3 NACHT domain and inhibits its interaction with NEK7. Experimental Autoimmune Encephalomyelitis pathogenesis (EAE) is a murine model of multiple sclerosis characterized by CD4 T cell-mediated inflammation and demyelination and is dependent on NLRP3 mediated IL-1β production. Manoalide attenuated clinical, pathological, and inflammatory features of EAE in mice, suggesting that manoalide can be used in pathologies associated with overt NLRP3 activation.

We thank the authors who contributed to this Research Topic wherein we highlight the function and significance of inflammasomes in immune system-related diseases. As exemplified by these studies, the pathological context defines the function of and therefore can predict the impact of therapies that target inflammasome activation. Thus, the function of inflammasome activation should be clearly defined for these therapeutic interventions to be effective. Further research is needed to find new inflammasome modulators and markers to help clinicians decide whether inhibition or activation of inflammasomes and downstream mediators would improve patient recovery.

